# Epidemiology of Acute Kidney Injury in the Intensive Care Unit

**DOI:** 10.1155/2013/479730

**Published:** 2013-03-21

**Authors:** James Case, Supriya Khan, Raeesa Khalid, Akram Khan

**Affiliations:** Division of Pulmonary and Critical Care, Oregon Health and Science University, 3181 SW Sam Jackson Park Road, UHN67, Portland, OR 97239-3098, USA

## Abstract

The incidence of acute kidney injury (AKI) in the intensive care unit (ICU) has increased during the past decade due to increased acuity as well as increased recognition. Early epidemiology studies were confounded by erratic definitions of AKI until recent consensus guidelines (RIFLE and AKIN) standardized its definition. This paper discusses the incidence of AKI in the ICU with focuses on specific patient populations. The overall incidence of AKI in ICU patients ranges from 20% to 50% with lower incidence seen in elective surgical patients and higher incidence in sepsis patients. The incidence of contrast-induced AKI is less (11.5%–19% of all admissions) than seen in the ICU population at large. AKI represents a significant risk factor for mortality and can be associated with mortality greater than 50%.

## 1. Introduction 

Acute kidney injury (AKI) (also referred to as acute renal failure) reflects a broad spectrum of clinical presentations ranging from mild injury to severe injury that may result in permanent and complete loss of renal function. The range of severity and variety of causes of AKI has resulted in multiple classification systems complicating diagnosis and subsequent management. The lack of consensus has resulted in a broad range of estimated prevalence in the intensive care unit (ICU) ranging from 1% to 70% depending on the criteria used [54, 55]. The underlying mechanisms of AKI include a decrease in the kidney's ability to excrete nitrogenous waste, manage electrolytes, regulate intravascular volume, and assist with maintenance of the acid-base status. The clinical effects of AKI depend on the clinical situation but almost invariably increase mortality and morbidity [56, 57]. 

## 2. Measurement of Renal Function

Definitions of AKI depend on measurement of serum creatinine (Cr) as a surrogate marker for the glomerular filtration rate (GFR). While inulin remains the gold standard for determination of GFR, it is rarely performed due to the need for continuous intravenous infusion and considerable laboratory resources [58]. Calculation of the GFR is performed based on the serum Cr value despite the fact that Cr has a complex metabolism and many factors can alter serum Cr values [59–64]. High protein intake and medications may increase Cr production independently of renal filtration. Furthermore, Cr secretion in the proximal renal tubules [65, 66] may account for as much as 60% of Cr elimination in patients with renal disease [67]. Medications such as cimetidine, quinidine, and trimethoprim inhibit this process [65], whereas hemodialysis can increase it [68]. Direct measurement of Cr clearance to determine renal function is also unreliable in critically ill patients due to the increased secretion of Cr in the renal tubules [67] and significant overestimation of GFR [58]. 

## 3. Defining AKI

Despite its first reports in the medical literature starting in 1917 described as “war nephritis” [69], AKI research was plagued by inconsistent definitions (as many as 35 different definitions [70]) until the Acute Dialysis Quality Initiative (ADQI) published the Risk, Injury, Failure, Loss, End-Stage (RIFLE) criteria in 2004 which defined AKI in terms of changes in serum Cr from baseline as well as urine output [1] (Table 1). Baseline Cr must be estimated in patients in which it is not known. Formulas estimating GFR may overestimate it in obese patients due to the overestimation of muscle mass. This can be alleviated by using the MDRD equation which incorporates body mass index to help nullify this effect [71]; however, estimation of baseline Cr values is still not reliable when used in ICU patients with AKI [72]. 

Subsequent research by Chertow et al. [57] suggested that even small changes in serum Cr were associated with increased mortality; so, the Acute Kidney Injury Network (AKIN) proposed more sensitive guidelines for the diagnosis of AKI [2] (Table 1). Multiple subsequent comparisons of sensitivity and prognosis of RIFLE and AKIN criteria have revealed conflicting results as to which method is superior although the majority of clinical data reflects RIFLE criteria [6, 17, 30, 56, 73–75]. 

## 4. Epidemiology of AKI 

Clinical studies assessing the exact incidence of AKI in the ICU (Table 2) proved sparse and were often complicated by differing criteria for the definition of AKI, especially prior to the adoption of the RIFLE and AKIN classifications. Furthermore, clinicians frequently underreport the incidence of AKI in their patients with one study showing that discharge summaries reported renal insufficiency in only 13% of affected patients [76]. Population-based analyses vary widely between 140 and 2880 cases per million population yearly with a 400% increase between 1988 and 2002 [77–81]. 

The incidence of AKI in hospitalized patients has also increased during the same time period from 4.9% in 1983 [82] to 7.2% in 2002 [83] to as much as 20% in 2012 [84]. This may partly be due to the definitions of AKI becoming more time sensitive and may reflect an increase in detection rather than an overall increase in incidence in disease. Due to the inconsistency of AKI definitions, the remainder of this paper will focus on articles published after release of the RIFLE criteria in 2004.

Many risk factors have been identified for the development of AKI including hypotension, pulmonary disease, liver failure, sepsis, hypovolemia, increased age, hypertension, preexisting renal disease, heart failure, bleach injection, and many medications (ACE inhibitors, vasopressors, aminoglycosides, NSAIDs, etc.) [17–21, 45, 85–93]. 

## 5. Causes of AKI

Causes of AKI are frequently categorized as prerenal, intrinsic renal, and postrenal. This classification system oversimplifies the overlapping pathologic mechanisms underlying AKI. Renal parenchymal tissue hypoperfusion due to hypovolemia or hypotension may initially cause a reversible increase in Cr. As cellular dysfunction continues, renal tubular cells may sustain ischemic injury which may persist after correction of the initial hypoperfusion state thereby changing from a prerenal azotemia to acute tubular necrosis. Rachoin et al. demonstrated that ICU patients with AKI and a blood urea nitrogen : Cr ratio greater than 20 : 1 had increased mortality suggesting that prerenal azotemia is not benign in ICU patients [94]. Acute tubular necrosis (ATN) is the most common form of intrinsic renal failure in the ICU comprising as much as 88% of all cases of AKI [95, 96]. Only one study describes postrenal AKI [97]. Two centers with a combined capacity of over 850 beds only identified 54 patients over a 13-year period suggesting a low overall incidence. Many causes of AKI in ICU patients likely represent multifactorial etiologies [96]. 

## 6. Contrast-Induced AKI

The administration of intravenous contrast prior to computed tomography (CT) scanning has been associated with the development of AKI. Overall incidence of contrast-induced AKI in ICU patients ranged from 11.5% to 19% [42–45] and mortality from 13.1% to 35.9%, much lower than reported in studies not involving intravenous contrast. Decreased mean arterial pressure, vasopressor usage, diuretics, increased age, and elevated baseline Cr are all associated with increased risk of AKI [42, 44]. 

## 7. Medical Patients

ICUs have seen an increase in admissions involving AKI ranging from 13% up to 78% [98–101]. Most studies have mixed surgical and nonsurgical populations. In 2002, Metnitz et al. published a series of ICU admissions comprised of over 17,000 patients (medical, surgical, and mixed ICUs) from 30 Austrian ICUs comprised of 50.4% medical admissions [102]. The primary endpoint defined as the need for renal replacement therapy (RRT) was noted in 4.9% of all ICU admissions (5.6% of medical ICU admissions and 4.5% of surgical ICU admissions). The most common reasons for admission that resulted in RRT were respiratory disease, sepsis, abdominal surgery, cardiovascular surgery, and surgery not otherwise specified.

## 8. Surgical Patients

Data from surgical populations show a similar incidence of postoperative AKI generally ranging from 16.7% to 30% [29, 103]. One study examining heart transplants revealed a 70% incidence of AKI in 54 patients, but this data may have been due to an increased acuity in both donors and recipients reflected as an overall mortality of 28% [55]. Roh et al. [35] reported 54% incidence of AKI in 98 patients following surgery for acute aortic dissection, 96% of which underwent emergent repair. Of these patients, 75% underwent moderate hypothermia during their procedure; however, hypothermia alone has not been shown to increase the incidence of AKI in thoracic aortic surgery [104]. Administration of intravenous contrast frequently precipitates AKI and also increases the risk of perioperative AKI beyond what would be expected from either surgery or percutaneous coronary intervention if both are performed during the same hospitalization, 33% versus 50% [32]. Few studies describe postoperative AKI in noncardiac/nonvascular patients. A Hungarian study examined 295 adults admitted to the ICU after major noncardiac surgery and found a similar 18.1% incidence of AKI [13]. However, 1129 patients undergoing lung resection surgery had an overall 5.9% incidence of AKI, 88% of which were classified as stage 1 by AKIN criteria [105]. 

## 9. Special Patient Populations

Multiple patient populations deserve special discussion regarding their risks for developing AKI. 

*Trauma*. Historically, trauma patients are younger than many other hospital populations, but as the median age of trauma patients increases, their incidence of AKI does not vary significantly from other ICU populations [106]. A post hoc analysis of the “Inflammation and the Host Response to Injury” dataset revealed a 26% incidence of AKI based on RIFLE criteria [107]. 

*Sepsis*. Severe sepsis, often complicated by renal hypoperfusion in septic shock, is characterized by a profound inflammatory milieu and associated dysfunction of multiple organ systems. Rates of AKI reported in septic patients range from 13% up to >64.4% [49–51, 87, 108, 109]. The toxic effects of sepsis on AKI do not appear to be specific to bacterial or fungal pathogens. The 2009 H1N1 influenza pandemic demonstrated that viral infections caused significant rates of AKI (up to 51%) sometimes exceeding those seen in more common infections [49–53]. Of note, delays in the administration of appropriate antibiotics are associated with the highest incidence of sepsis-induced AKI. A 6.0 h versus 4.3 h delay in antibiotics resulted in an odds ratio of 1.14 (95% CI 1.10–1.20, *P* < 0.001) per hour delay for the development of AKI [87]. Furthermore, current sepsis resuscitation guidelines advocate aggressive volume resuscitation for patients presenting with severe sepsis in an effort to improve overall survival [110]. Little data exists describing the renal effects of this resuscitation; however, one study demonstrated an increase in AKI developing within the first 2 days of ICU admission in those patients with a positive fluid balance [111]. Payen et al. performed a post-hoc analysis of the Sepsis Occurrence in Acutely Ill Patients (SOAP) trial to assess the effects of fluid resuscitation on the development of AKI. Septic patients in the SOAP trial received significantly more fluid than their nonseptic counterparts during the first 72 hours of ICU admission (1.8 ± 5.0 versus 0.51 ± 3.5 L). Early, aggressive volume resuscitation can improve outcomes [112, 113]; however, patients with a more positive fluid balance subsequently developed AKI more frequently than patients that had a less positive balance [114] suggesting that the benefits of fluid administration may be time sensitive. 

*H1N1*. Little data regarding the incidence of AKI in patients with influenza A infection had been published prior to the H1N1 pandemic in 2009. Multiple case reports of rhabodmyolysis exist, but no systematic analyses of incidence have been published [115–119]. Therefore, it is impossible to determine whether the H1N1 strain uniquely caused AKI or whether the many reports of AKI reflect the underlying severity of the patients' illness upon their arrival in the ICU [49–53]. 

*Burns*. Victims of severe burns often experience a similar massive inflammatory response but without the underlying infectious toxins as many septic patients. In addition, these patients often have an exact time of onset, often immediately prior to presentation, and receive immediate resuscitation as opposed to septic patients that may develop symptoms over hours to days prior to presentation and initiation of therapy. Despite this early, aggressive therapy, burn patients experience AKI as often as sepsis patients. In a study of 60 patients admitted with burns exceeding 20% body surface area, 53.3% developed AKI [24]. This reflects the upper range of AKI seen in the literature; however, the mortality for AKI in burn patients is somewhat lower than expected (7.6–34.4%) [22–24]. 

*Cardiac Arrest*. Patients experiencing cardiac arrest often have prolonged periods of severe hypotension. Of those that survive and achieve return of spontaneous circulation (ROSC), the need for vasoactive medications beyond 24 hours after ROSC correlates with the development of AKI, 51.7% versus 6.4%, likely reflecting overall systemic injury [120]. 

*Trauma*. The incidence of AKI in trauma patients trends similarly to other populations, but the mortality did not reach levels seen in other studies. This may reflect the younger patient populations seen by Gomes' (mean age = 37) mortality rate of 8.2% [27] compared to Constantini's (mean age = 42.3 with AKI and 52.9 without AKI) mortality rate of 10.8% [26] and Bagshaw's (mean age = 62.5 with AKI and 47.3 without AKI) [17]. 

*Cardiothoracic and Vascular Surgery*. Patients undergoing cardiac surgery face a unique combination of renal stressors from both major surgery as well as cardiopulmonary bypass. Rates of AKI for elective cases generally range from 16.7% to 32.8% with a concomitant low mortality (1.3%–5.5%) with 2 exceptions [35, 36]. In 817 patients undergoing coronary artery bypass grafting while being on cardiopulmonary bypass, the incidence of AKI was 48.5% and mortality 12.6% [36]. Some of the decreased AKI and mortality rates may be explained by patient selection prior to undergoing surgery, but Roh et al. published a series of patients undergoing thoracic aortic graft placement of which 95% of the cases were performed emergently suggesting that patient selection alone may not be the sole factor [35]. The increased incidence of AKI and mortality noted by Lin et al. most likely reflects the prolonged duration of bypass since it was used primarily for the treatment of postcardiotomy cardiogenic shock well after completion of the surgical procedure [28]. Of note, vascular surgery in itself does not appear to be a major risk factor for AKI mortality [41]. 

## 10. Conclusions 

AKI in the ICU has been difficult to study due to varying criteria for the definition of it prior to the adoption of the RIFLE and AKIN classifications. There has been a gradual increase in incidence of AKI which may partly be explained by an increase in detection. Furthermore, clinicians frequently underreport the incidence of AKI in sensitivity of the criteria to define AKI. The overall incidence of AKI in the ICU is approximately 20%–50% and can have a mortality over 50%. With the exception of liver transplant patients, surgical patients generally have a lower incidence of AKI, whereas patients with sepsis have the highest incidence. Future studies may benefit by better identifying modifiable risk factors to prevent the development of AKI.

## Figures and Tables

**Table 1:** Definitions of AKI.

	Serum Cr criteria	Urine output criteria
RIFLE [1]		
R (Risk)	Increase in serum Cr × 1.5 or GFR decrease >25%	<0.5 mL/kg/h for 6 hours
I (Injury)	Increased serum Cr × 2 or GFR decrease >50%	<0.5 mL/kg/h for 12 hours
F (Failure)	Increase in serum Cr × 3, GFR decrease >75%, or serum Cr > 4 mg/dL	<0.3 mL/kg/h for 24 hours or anuria for 12 hours

AKIN [2]		
Stage 1	Increase in serum Cr of ≥0.3 mg/dL or increase to ≥150–200% from baseline	<0.5 mL/kg/h for more than 6 hours
Stage 2	Increase in serum Cr to >200–300% from baseline	<0.5 mL/kg/h for more than 12 hours
Stage 3	Increase in serum Cr to >300% from baseline (or serum Cr ≥ 4.0 mg/dL with an acute increase of ≥0.5 mg/dL)	<0.3 mL/kg/h for 24 hours or anuria for 12 hours

**Table 2:** Summary of original reports describing the incidence of AKI.

Author	no. of subjects	Single/multicenter	Patient population	AKI definition used	AKI incidence (%)	Mortality endpoint	Mortality (%)
Thakar et al. [3]	325,395	Multi	Mixed	RIFLE	22	ICU	10.9
Ostermann an Chang [4]	41,972	Multi	Mixed	RIFLE	35.8	ICU Hospital	10.2 12.9
Gammelager et al. [5]	30,762	Multi	Mixed	RIFLE	15.6	30 days	40
Joannidis et al. [6]	16,784	Multi	Mixed	RIFLE	35.5	Hospital	36.4
Mandelbaum et al. [7]	14,524	Single	Mixed	AKIN	57	ICU Hospital	7.1 9.1
Hoste et al. [8]	5383	Single	Mixed	RIFLE	67.2	Hospital	13.3
Cruz et al. [9]	2164	Multi	Mixed	RIFLE	10.8	ICU	36.3
Samimagham et al. [10]	1026	Single	Mixed	RIFLE	21.7	1 year	49 2.5 RRT at 90 d
Fonseca Ruiz et al. [11]	794	Single	Mixed	AKIN	39.8	ICU Hospital	25.4 32.1
Piccinni et al. [12]	576	Multi	Mixed	RIFLE	42.7	ICU	29
Medve and Gondos [13]	459	Single	Mixed	AKIN	24.4	ICU Hospital	39.3 49.1
Samimagham et al. [10]	235	Single	Mixed	AKIN	31.1	ICU	72.6
Yue et al. [14]	191	Single	Mixed	AKIN	35.5	ICU	48
Abosaif et al. [15]	183	Single	Mixed	RIFLE		ICU	47.5
Yegenaga et al. [16]	139	Single	Mixed	RIFLE	56.8	UKN	37.4
Bagshaw et al. [17, 18]	120,123	Multi	Sepsis	RIFLE versus AKIN	36.1 37.1	Hospital	RIFLE: 24.2 AKIN: 24.5
Kim et al. [19]	291	Single	Severe sepsis/ Septic shock	RIFLE versus AKIN	RIFLE: 62.9 AKIN: 65.6	28 days	RIFLE: 58.5 AKIN: 57.6
Lopes et al. [20]	182	Single	Sepsis	RIFLE	37.4	NS	37.4
Lerolle et al. [21]	35	Single	Septic shock	RIFLE	65.8	28 days	62.8
Coca et al. [22]	304	Single	Burn	RIFLE	26.6	NS	7.6
Lopes et al. [23]	126	Single	Burn	RIFLE	35.7	NS	17.5
Palmieri et al. [24]	60	Single	Burn	RIFLE	53.3	UKN	34.4
Bagshaw et al. [25]	9449	Multi	Trauma	RIFLE	18.1	Hospital	16.7
Costantini et al. [26]	571	Single	Trauma	AKIN	29.8	Hospital	15.9
Gomes et al. [27]	436	Single	Trauma	RIFLE	50	ICU Hospital	8.2 10.8
Lin et al. [28]	46	Single	ECMO	RIFLE	78	Hospital	60.9
Englberger et al. [29]	951	Single	Tricuspid valve surgery	RIFLE	30	30 days	5.5
Englberger et al. [30]	851	Single	Thoracic aortic surgery	RIFLE	17.7	30 days	1.3
Kuitunen et al. [31]	813	Single	Cardiac surgery	RIFLE	19.3	90 days	3.2
Kramer et al. [32]	668	Multi	Cardiac surgery	AKIN	Surgery: 33.7 Cath + surgery: 50.2	NS	NS
Yan et al. [33]	509	Single	Cardiac surgery	RIFLE	32.8	Hospital	4.3
Mariscalo et al. [34]	414	Multi	Aortic root repair	RIFLE	16.7	Hospital	2.7
Roh et al. [35]	98	Single	Thoracic aorta graft placement	RIFLE	54	30 days	5.1
Machado et al. [36]	817	Single	CP bypass	RIFLE	48.5	30 days	12.6
Sirvinskas et al. [37]	179	Single	CP bypass	RIFLE	10.6	NS	NR
O'Riordan et al. [38]	300	Multi	OLT	RIFLE	36.8	NS	NS
Biagioni et al. [39]	144	Single	OLT versus NEAS	RIFLE	OLT: 29 NEAS: 47	ICU	OLT: 29 NEAS: 51
Guitard et al. [40]	97	Single	OLT	RIFLE	63.8	UKN	UKN
Medve and Gondos [13]	295	Multi	Major noncardiac surgery	AKIN	18.1	ICU	33.3
Tallgren et al. [41]	69	Single	Elective infra-renal AAA surgery	RIFLE	22	Hospital	1.4
Hoste et al. [42]	787	Single	CIN	UKN	16.3	28 days 1 year	13.1 35.9
Lakhal et al. [43]	299	Single	CIN	AKIN	14	ICU	18
Rashid et al. [44]	139	Single	CIN	RIFLE	11.5	ICU	31
Valette et al. [45]	101	Single	CIN/Surgical	RIFLE versus AKIN	RIFLE: 19 AKIN: 19	ICU	RIFLE: 26.3 AKIN: 31.6
Chua et al. [46]	105	Single	ROSC following cardiac arrest	RIFLE	PRCS: 51.7 No PRCS: 6.4	ICU	PCRS: 46.6 No PCRS: 27.7
						Hospital	PCRS: 51.7 No PCRS: 34
Cholongitas et al. [47]	412	Single	Cirrhosis	RIFLE	50	6 weeks	61.2
Hata et al. [48]	376	Single	ADHF	RIFLE	73.1	Hospital	10.5
Martin-Leoches et al. [49]	661	Multi	H1N1	AKIN	17.7	ICU	44.1
Jung et al. [50]	221	Multi	H1N1	RIFLE	22.6	30 days	17.2
Nin et al. [51]	84	Multi	H1N1	RIFLE	51	28 days	51.2
Abdulkader et al. [52]	47	Single	H1N1	RIFLE	53	Hospital	19.1
Chacko et al. [53]	31	Single	H1N1	RIFLE	3.2	28 days	16.1

Mixed: combined medical and surgical patients. RRT: renal replacement therapy. ECMO: extracorporeal membrane oxygenation. OLT: orthotopic liver transplant. NEAS: non-emergent abdominal surgery. CP: cardiopulmonary. NS: not specified. UKN: unable to determine due to inability to access source. AAA: abdominal aortic aneurysm. CIN: contrast-induced nephropathy. ROSC: return of spontaneous circulation. PRCS: post-resuscitation cardiogenic shock. ADHF: acute decompensated heart failure.
